# Radioresistance in rhabdomyosarcomas: Much more than a question of dose

**DOI:** 10.3389/fonc.2022.1016894

**Published:** 2022-09-29

**Authors:** Simona Camero, Matteo Cassandri, Silvia Pomella, Luisa Milazzo, Francesca Vulcano, Antonella Porrazzo, Giovanni Barillari, Cinzia Marchese, Silvia Codenotti, Miriam Tomaciello, Rossella Rota, Alessandro Fanzani, Francesca Megiorni, Francesco Marampon

**Affiliations:** ^1^ Department of Maternal, Infantile and Urological Sciences, Sapienza University of Rome, Rome, Italy; ^2^ Department of Radiological Sciences, Oncology and Anatomical Pathology, Sapienza University of Rome, Rome, Italy; ^3^ Department of Oncohematology, Bambino Gesù Children’s Hospital, Istituto di Ricovero e Cura a Carattere Scientifico (IRCCS), Rome, Italy; ^4^ Department of Clinical Sciences and Translational Medicine, University of Rome Tor Vergata, Rome, Italy; ^5^ Department of Oncology and Molecular Medicine, Istituto Superiore di Sanità, Rome, Italy; ^6^ Units of Molecular Genetics of Complex Phenotypes, Bambino Gesù Children’s Hospital, Istituto di Ricovero e Cura a Carattere Scientifico (IRCSS), Rome, Italy; ^7^ Department of Experimental Medicine, “Sapienza” University of Rome, Rome, Italy; ^8^ Department of Molecular and Translational Medicine, University of Brescia, Brescia, Italy

**Keywords:** rhabdomyosarcoma, radiotherapy, radiation therapy, radioresistance, radiosensitizers

## Abstract

Management of rhabdomyosarcoma (RMS), the most common soft tissue sarcoma in children, frequently accounting the genitourinary tract is complex and requires a multimodal therapy. In particular, as a consequence of the advancement in dose conformity technology, radiation therapy (RT) has now become the standard therapeutic option for patients with RMS. In the clinical practice, dose and timing of RT are adjusted on the basis of patients’ risk stratification to reduce late toxicity and side effects on normal tissues. However, despite the substantial improvement in cure rates, local failure and recurrence frequently occur. In this review, we summarize the general principles of the treatment of RMS, focusing on RT, and the main molecular pathways and specific proteins involved into radioresistance in RMS tumors. Specifically, we focused on DNA damage/repair, reactive oxygen species, cancer stem cells, and epigenetic modifications that have been reported in the context of RMS neoplasia in both *in vitro* and *in vivo* studies. The precise elucidation of the radioresistance-related molecular mechanisms is of pivotal importance to set up new more effective and tolerable combined therapeutic approaches that can radiosensitize cancer cells to finally ameliorate the overall survival of patients with RMS, especially for the most aggressive subtypes.

## Introduction

### Rhabdomyosarcoma

**Figure 1:**
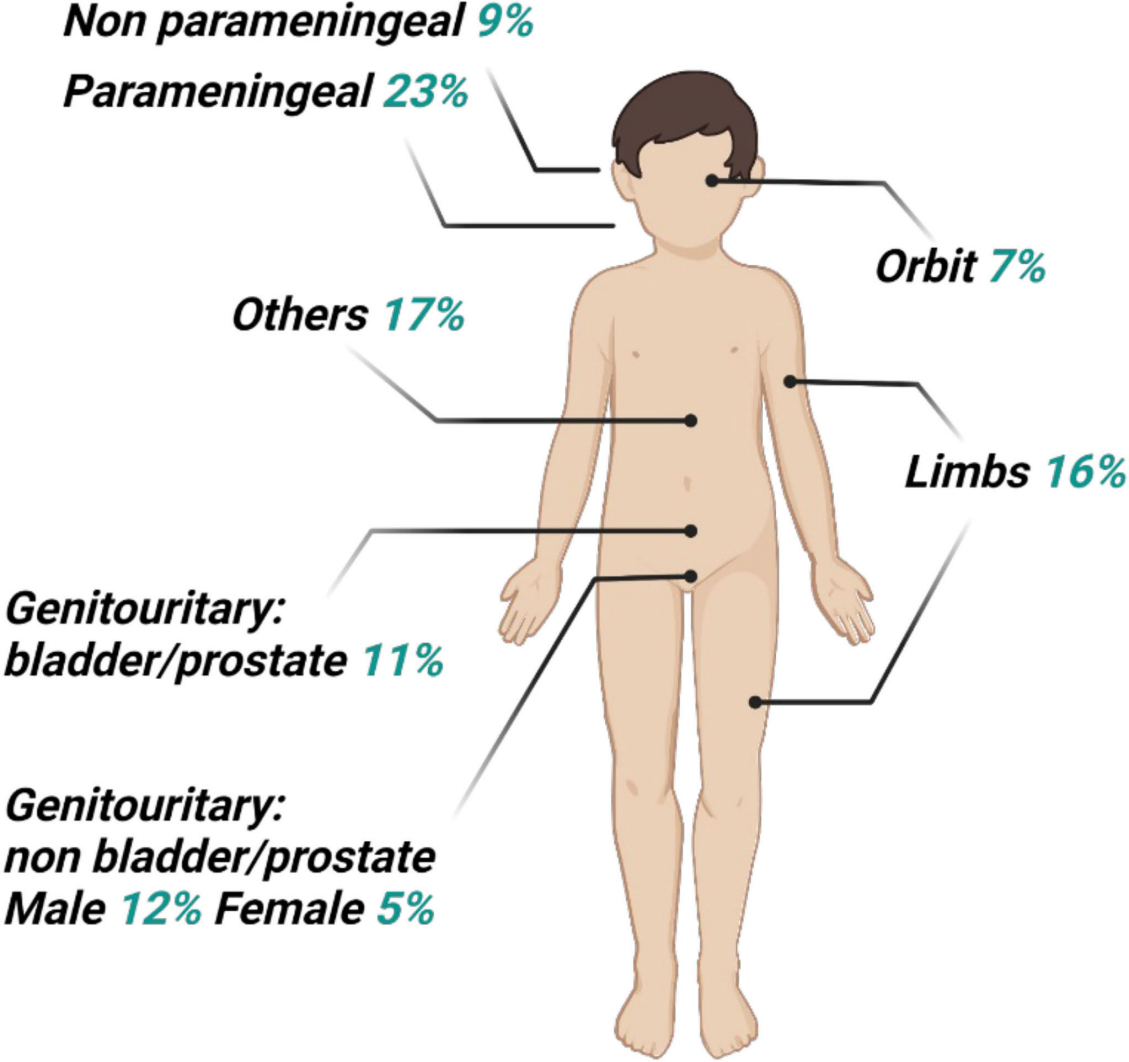
Distribution of primary sites for rhabdomyosarcoma. The head and neck site may be subdivided as 7% orbit, 8% other head, 23% parameningeal, and 9% non-parameningeal. The pelvic sites may be subdivided as 11% bladder and prostate, and 5% female genital or 12% male non-bladder/prostate.

Rhabdomyosarcoma (RMS) is a highly aggressive soft tissue sarcoma (STS) that primarily affects pediatric patients, accounting for 5% of all childhood cancers and representing 3% of STS in adult, for whom it has a worse prognosis. As shown in **Figure 1**, the most common RMS location is in the head and neck region (35%–40%), genitourinary tract (bladder/prostate, 11%), genitourinary tract non-bladder/prostate (male, 12%; female, 5%), and limbs (16%). Signs and symptoms at presentation will depend on the site of the primary tumor, whether there is extension into contiguous organs, and, in some cases, the presence of metastatic disease (1). Originally, two major subtypes of RMS were recognized: embryonal RMS (ERMS), preferring male children, and alveolar RMS (ARMS), which remains constant throughout childhood and adolescence, showing the worse prognosis. Others two rarer RMS subtypes are the pleomorphic RMS and the spindle cell/sclerosing RMS, which typically occur in adults and children, respectively (2, 3). ARMSs more frequently carry t (2;13)(q35;q14) or t(1;13)(p36;q14) chromosomal translocations that, juxtaposing Paired box gene 3 (PAX3) on chromosome 2 or PAX7 on chromosome 1 with Forkhead box protein O1 (FOXO1) on chromosome 13, generate PAX3–FOXO1 and PAX7–FOXO1 fusion genes, respectively, and finally transcribe/translate into pro-oncogenic fusion proteins with an aberrantly enhanced transcriptional activity (4, 5). Because fusion protein presence correlates with a poorer prognosis, nowadays, the preferred RMS classification is expressing, i.e., “fusion positive” (FP-RMS), or not expressing fusion protein, i.e., “fusion negative” (FN-RMS) (6). ERMSs (FN-RMS tumors), more frequently present various mutations largely converging on a limited number of pathways, also perturbed in FP-RMSs, indicating some commonality in the molecular driving forces in RMS (2). FN-RMSs often harbor a mutation affecting mitogen-activated protein kinases and/or PI3K–AKT–mTOR pathways (7, 8), aberrantly activated also in FP-RMS, to the ability of fusion proteins to activate several cell surface receptor tyrosine kinases upstream of these pathways (5). Notably, patients with FN-ARMS are clinically and molecularly indistinguishable from ERMS (9).

This review presents a brief overview of the guidelines for the diagnosis and treatment of RMS, with particular emphasis on the role of radiotherapy (RT) and on the molecular mechanisms mainly responsible for radioresistance, focusing on possible candidate radiosensitizing strategies in RMS. In particular, after summarizing the key principles of the management of the patient with RMS, from diagnosis to treatment, focusing on the role of RT and on the novelties in terms of indications, therapy schemes, and treatment techniques, we will analyze the principles of radiobiology and of the RMS and, therefore, the molecular mechanisms of radioresistance.

### RMS diagnosis and staging

**Figure 2:**
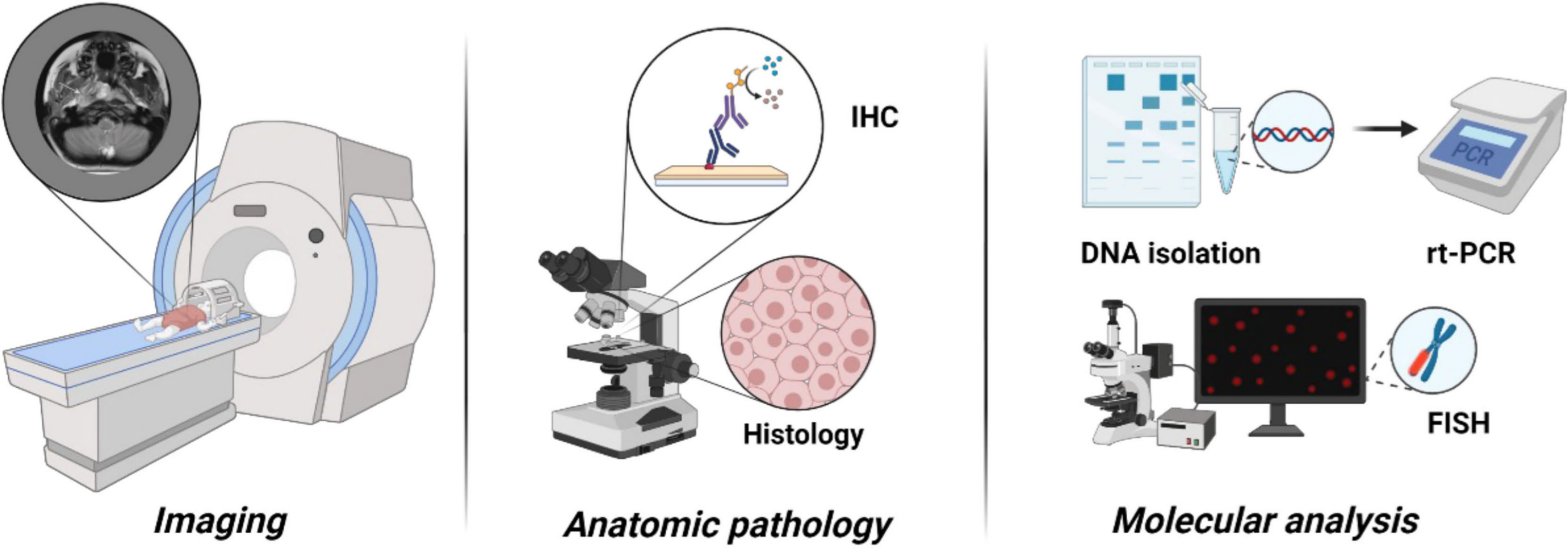
The role of molecular analysis for differential diagnosis and classification of RMS. Molecular analysis helps to identify the subtypes and classify RMS.

After the head and neck site (35%–50%), the genitourinary tract represents the primary site for around 20%–25% of RMS pediatric patients, resulting as exceedingly rare in adults (10, 11). Vagina or uterus, favorable sites, are more frequently involved, followed by the kidney, bladder, or prostate, considered as unfavorable sites (12, 13). At the onset, hematuria and urinary obstruction represent the signs and symptoms more frequent, whereas 10%–20% of pediatric and 40% of adult patients present distant metastases. RMS diagnosis requires, in addition to standard laboratory (complete blood counts, electrolytes, renal function tests, liver function tests, and urinalysis), the direct evaluation of tumor tissue derived from either an incisional/excisional biopsy or a core needle biopsy (1) magnetic resonance imaging, for local staging, and computed tomography (CT) and [F-18]2-fluoro-2-deoxyglucose positron emission tomography (18F-FDG PET)/CT), for systemic staging and the risk stratification (14, 15). TNM staging is based on the anatomic location and invasiveness of the primary tumor, tumor size, nodal status, and extent of metastasis (**Tables 1**, **2**). Intergroup Rhabdomyosarcoma Study Group (IRSG) establishes risk group stratification, identifying low-, standard-, high, or very high–risk patients. The clinical subgroup is primarily determined by IRSG group, lymph node involvement, fusion protein expression, site, and age (16, 17). Risk stratification is summarized in **Table 3**. The bioptic samples must be subjected to a series of histology and molecular pathology studies aimed at measuring myogenic markers like desmin, skeletal alpha-actin, myosin, and myoglobin and early myogenesis transcription factors like MyoD and myogenin (18). The analysis of these markers is achieved with immunohistochemical assays, and it is combined with cell morphological assessment in light microscopy, used to distinguish RMS from other childhood neoplasms also expressing myogenic proteins (18). More recently, molecular analysis has become an essential tool for differential diagnosis and classification of RMS (**Figure 2**). Specifically, Real-Time PCR (RT-PCR) and Fluorescence in situ hybridization (FISH) assays designed to measure the expression of fusion gene PAX3–FOXO1 or PAX7–FOXO1 are very useful to identify subsets of ARMS, and microarray genome-wide RNA expression techniques have been shown to generate, through various statistical algorithms, “diagnostic signatures” of the FP-RMS and FN-RMS categories (2).

**Table 1:** TNM classification for rhabdomyosarcoma.

T: Tumor Stage		
T_1_: Confined to anatomic site of origin	T_1a_: ≤5 cm	T_1b_: >5 cm
T_2_: Extension and**/**fixative to surrounding tissue	T_1a_: ≤5 cm	T_1b_: >5 cm
**N: Regional Nodes**		
N_0_: Not clinically involved	N_1_: Clinically involved	N_X_: Clinical status unknown
**M: Metastases**		
M_0_: No distant metastases	M_1_: Distant metastases present	

**Table 2:** TNM stage for rhabdomyosarcoma.

stage	Primary site	TNM stage	Tumor size	Regional nodes	Distant metastasis
1	Favorable*	T_1_ or T_2_	Any size	N_0_ - N_1_ - N_x_	M_0_
2	Unfavorable	T_1_ or T_2_	≤5 cm	N_0_ - N_x_	M_0_
3	Unfavorable	T_1_ or T_2_	≤5 cm	N_1_	M_0_
			>5 cm	N_0_ - N_1_ - N_x_	
4	Any	T_1_ or T_2_	Any size	N_0_ - N_1_ - N_x_	M_1_

*Favorable sites: orbit; non-parameningeal head and neck; genitourinary tract other than kidney, bladder, and prostate; and biliary tract.

**Table 3:** European Paediatric Soft Tissue Sarcoma Study Group staging of rhabdomyosarcoma.

Risk Group	Subgroups	FP (+)FN (-)	IRS Group		Site	Node Stage	Age / Size
			I = R0 or Complete		Favorable	N0	Favorable<10y / <5cm
			II = R1 or Microscopic disease or primary complete resection but N1				
			III = R2 or Macroscopic Disease		Unfavorable	N1	Unfavorable>10y / >5cm
			IV = Distant Metastases				
Low	A	–	I	R0	Any	N0	A(F)+S(F)
Standard	B	–	I	R0	Any	N0	A(F) or S(F)
Standard	C	–	II	R1	Favorable	N0	Any
			III	R2	
High	D	–	II	R1	Unfavorable	N0	Any
			III	R2	
High	E	–	II	R1	Any	N1	Any
			III	R2	
High	F	+	I	R0	Any	N0	Any
			II	R1	
			III	R2	
Very High	G	+	II	R1	Any	N1	Any
			III	R2	
Very High	H	Any	IV	Metastases	Any	Any	Any

### RMS treatments

Treatment of locally and locally advanced RMS is mainly based on surgery (14, 15), although, aggressive surgery, often necessary to achieve tumor debulking and negative microscopic margins, is no longer recommended (19). This is particularly true for genitourinary RMS, to avoid significant long-term morbidities such as urinary diversion, infertility, and sexual dysfunction particularly. Therefore, except for paratesticular tumors (20), the standard care of RMS, genitourinary and non-genitourinary, usually provides neoadjuvant chemotherapy (CHT) followed by RT or concomitant CHT/RT followed or not by excision (14, 15). In 20% of patients with genitourinary RMS, a close follow-up with imaging is a reasonable alternative to aggressive surgery (14, 15). In support of a delayed surgery, it has been shown that, despite RMS can persist after a neoadjuvant approach (21, 22), the rhabdomyoblasts found in subsequent biopsies progressively decrease and their presence do not necessarily predict local recurrence (23, 24). Thus, aggressive surgical resection, followed or not by RT, is usually performed for recurrent and metastatic RMS (25, 26). CHT is based on IVA (ifosfamide, vincristine, and actinomycin D) or VAC (vincristine, actinomycin D, and cyclophosphamide), respectively used, with no difference, in Europe or in North America (27, 28). Combining doxorubicin improves IVA, inducing several treatment-related adverse events (29). Low-dose maintenance CHT has been shown to improve outcome (30). Trabectedin is commonly used as a second line (31), whereas several clinical trials conducted to test the effects of several molecular targeted drugs combined or not with other targeted therapies or CHT had not shown significant clinical improvement (32–40). However, because of CHT-induced toxicities, pharmacological treatments are often interrupted (41).

### Current status of radiation treatment in RMS

Achieving local tumor control with the first-line treatment is crucial for patients with RMS (42). RT plays an increasingly critical role in the management of RMS for local control both at primary and metastatic sites and continues to be a major treatment modality for genitourinary RMS (43–49). Because routine use has been encouraged by the EpSSG RMS 2005 study (44), RT has been shown to improve the event-free survival rate of patients, whereas local failures have been shown to be more frequent when the irradiation is omitted (50). The dose, duration, and timing of external beam radiation therapy, in which x-rays can penetrate deeper in the tissues while minimizing skin irradiation and side effects (51), depend on the patient’s age, RMS type and histology, the site of origin of the tumor, how much tumor remained after surgery, and the local lymph nodes involvement. **Table 4** resumes treatment schedules (15, 52, 53). In case of adult patients with RMS, RT can provide a total dose from 50 up to 70 Gy, conventionally delivered (54–56). The use of hyperfractionated (hFRT) regimen, smaller doses per single fraction, performed by the large, randomized IRS-IV study, failed (57). This failure, however, has permitted to revise the radiobiology of RMS, as later discussed. On the other hand, the use of hyperfractionated (HFRT) regimen, larger doses per single fraction, delivered in combination or not with CHT, did not give significant advantages (58–70). Thus, the RMS response to RT appears to go far beyond the simple dose problem because, as for other highly radioresistant tumor types, RMS appears capable of activating a complex biological response supporting radioresistance.

**Table 4:** Doses and fractions of radiotherapy for patients over 3 years of age.

Conventional Radiotherapy (Age > 3 years)		
**IRS Group**	**ERMS**	**ARMS**
**I = R0 or Complete**		
**II = R1 or Microscopic Disease**		
**III = R2 or Macroscopic Disease**		
**I**	No radiotherapy	41.4 Gy in 23 fractions
**II**	41.4 Gy in 23 fractions	41.4 Gy in 23 fractions
**III**	50.4 Gy in 28 fractions	50.4 Gy in 28 fractions
**III → R0 after reoperation**	36 Gy in 20 fractions	41.4 Gy in 23 fractions
	41.4 Gy in 23 fractions	
**Complete clinical response to Chemotherapy and no surgery**	41.4 Gy in 23 fractions	50.4 Gy in 28 fractions
**Partial clinical response to Chemotherapy and no surgery**	45 Gy in 25 fractions	50.4 Gy in 28 fractions + boost 5.4 Gy in 3 fractions
**Stable clinical response to Chemotherapy and no surgery**	50.4 Gy in 28 fractions + boost 5.4 Gy in 3 fractions	50.4 Gy in 28 fractions + boost 5.4 Gy in 3 fractions
**Orbital**	45 Gy in 25 fractions	

## The radiobiology of RMS: The linear quadratic model and the question of dose

Radiobiology has been classically focused on achieving the greatest possible difference between a high probability of local tumor control [tumor control probability (TCP)] and a low risk of normal tissue complications [normal tissue complication probability (NTCP)], namely, therapeutic window. In fact, whether increasing the dose improves TCP, because of the lack of technology able to spare normal tissues, it also increases NTCP. Thus, RT has been long delivered by using daily fractions of 1.8–2.2 Gy, the conventional fractionation, which is still largely used today. The reason why conventional fractionation guarantees the best therapeutic window depends on the concept that normal cells repair sublethal damages more efficiently than cancerous cells, as shown from the linear quadratic model (LQ) (71) and from of “4Rs” of radiobiology (72). Briefly, LQ is a mathematical model describing the relationship between cell survival and delivered dose, and it is represented by the equation *S* = *e* ^−^ (71). The probability to survive (*S*) of a cell/tissue type to a single dose of radiation depends on the ratio between two factors: i) the number of cells directly killed by double-strand breaks (DSBs), namely, a; and ii) the number of cells that, having saturated the repair mechanisms, die for the accumulation of sublethal unrepaired single-strand breaks (SSBs), namely, b. The a/b ratio indicates the fraction size sensitivity of a tissue, with b indicating the ability of cell to repair SSBs. Hence, cells with a low a/b ratio efficiently repair SSBs, contrary to cells with a high a/b ratio (71). Notably, doses of RT close to 1.8–2.2 Gy induce thousands of repairable SSBs and few DSBs (73–75), thus indicating that the proportion of cells surviving to conventional fractionation strictly depends on the ability to repair SSBs. Thus, because cancer cells less efficiently “R”epair SSBs than normal cells, cancer cells slower “R”edistribute cell cycle from RT-induced G_2_/M arrest, less efficiently “R”epopulate killed cancer cells, and result more affected by the “R”eoxygenation of the central portions of the tumor induced by the progressive reduction of the peripheral regions. Those are the “4Rs” of radiobiology, historically supporting the efficiency of the conventional fractionation (72). However, after a long time, it was shown that not all cells within cancer population and not all patients with the same tumor have the same sensitivity to RT, introducing the fifth “R”, the “R”adiosensitivity (76). Thus, considering the technological evolution of RT that nowadays permits the safety delivery of larger fractions (77), the use of HFRT or Stereotactic Body Radiation Therapy (SBRT), ablative dose of radiation, has been proposed as strategy to overcome the intrinsic radioresistance of cancer. Furthermore, this choice is also supported by the fact that increasing evidence shows the ability of HFRT and SBRT to “R”eactivate the anti-tumor immune response, the sixth “R” of radiobiology (78). As previously discussed, the use of higher dose per fraction has been also proposed for RMS as a consequence of the low a/b ratio (2.8 Gy) shown for this cancer type (79). However, the use of HFRT for the treatment of RMS did not lead to any improvement in efficacy (58–70), as already described for other cancer types (80–84). Despite being a milestone in the multimodal treatment of pediatric RMS, RT is still significantly associated with local failure in most cases of tumor relapse, and the RMS response to radiation appears to go far beyond the simple dose problem. Indeed, together with the development of more sophisticated and effective technologies, overcoming radioresistance seems to be not just a question of dose but rather of understanding the cellular mechanisms that support radioresistance to identify future radiosensitizing strategies.

## Mechanisms of radioresistance in RMS

**Figure 3:**
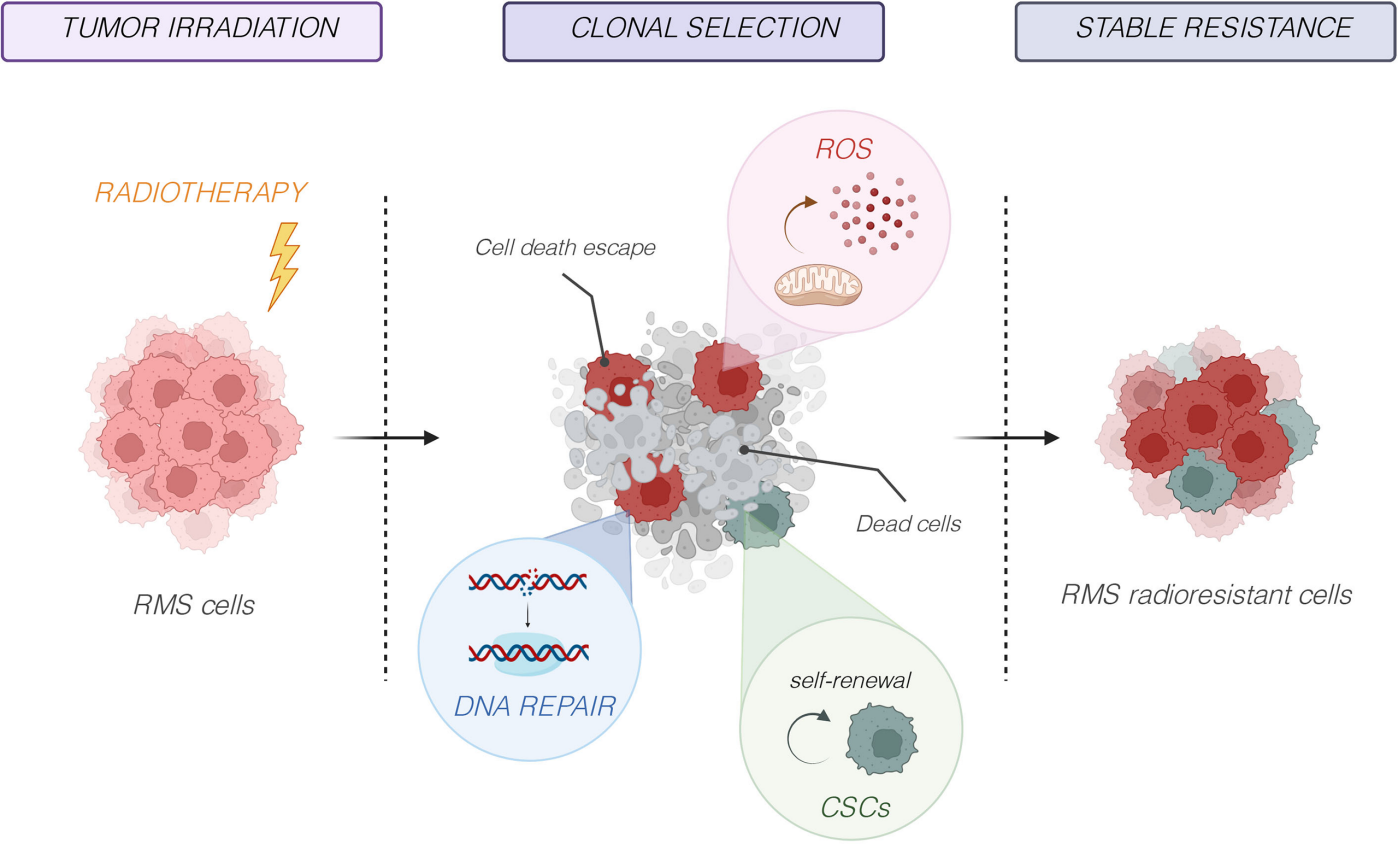
Molecular mechanisms responsible of radioresistance. Several key cellular and molecular factors, including DNA damage and repair, oxidative stress, tumor microenvironment, cancer stem cells (CSCs), and tumor heterogeneity, are implied in RMS radioresistance.

As other highly radioresistant tumor types, RMS appears capable of activating a complex biological response that makes them capable of resisting even high radiation doses. Therefore, it is necessary to deeply elucidate the precise mechanisms that are responsible for the radioresistance in RMS to identify new radiosensitizing therapeutic strategies. Over the last years, different studies have identified several key cellular and molecular factors, including DNA damage and repair, oxidative stress, tumor microenvironment, cancer stem cells (CSCs), and tumor heterogeneity (**Figure 3**), which are implied in RMS radioresistance and are discussed in detail in the following subsections.

### RT and DNA breaks/damage response-related pathways

Ionizing radiations used in RT are electrically charged particles, which deposit energy in the tissues that they pass through, killing cancer cells or causing genetic changes that lead to cancer cell death (85). On a cellular level, the biological target of radiation is DNA, by inducing several types of DNA damages involving one strand (SSBs) or both strands of DNA (DSBs) (86, 87). Although radiations damage both cancer and normal cells, cancer cells are generally less efficient in repairing damages caused by RT, resulting in differential killing outcomes (88). DSBs, the most lethal form of DNA damage and a primary cause of cell death induced by RT, can be divided in simple and complex types. Simple DSBs are two-ended breaks of DNA, usually directly consequent to the action of radiation, whereas complex DSBs are clusters of different DNA damages including single-base mutations, insertions, and deletions and/or SSBs around DSBs, generally indirectly induced by radiation through the production of reactive oxygen species (ROS) (89–93). Thus, contrary to SSBs and simplex DSBs, complex DSBs are usually inefficiently repaired, determining genomic instability and cell death (94–96) although cancer cells can activate specific DNA damage repair mechanisms, thus surviving the following irradiation (92). The homologous recombination (HR) and the non-homologous end joining (NHEJ) mechanisms represent the most prominent pathways, orchestrating the DNA damage response (DDR) in eukaryotic cells. HR uses homologous sequences of undamaged sister chromatid as a template to repair DSBs, thus resulting in an error-free DDR mechanism (97). HR is mainly regulated by the MRN complex (Mre11–Rad50–Nbs1), which recognizes DSBs recruiting activated ataxia-telangiectasia mutated (ATM) that, in turn, orchestrates the activity of Breast Cancer gene 1 (BRCA1), Breast Cancer gene 2 (BRCA2), Checkpoint kinase 2 (CHK2), RAD51, and tumor protein 53 (p53)—key factors involved in HR. Parallelly, ATR (ataxia-telangiectasia mutated and Rad3-related) kinase attenuates DSB-induced ATM activation by switching DSB ends from an ATM-activating mode to an ATR-activating model (98) and activates CHK1 that slows or arrests cell cycle progression, thus allowing more time for DNA repair (99). Mutation inactivating BRCA1 and/or BRCA2 and/or other(s) gene(s) of the HR pathway, namely, the “BRCAness” status, permits to stratify HR-deficient (HRD) from HR-proficient (HRP) cancers (100) and to identify HRD as more sensitive to “synthetic lethality” mediated by PARP inhibitor (PARPi) (101, 102). The “BRCAness” phenotype has been shown in several types of sarcomas (103–105), including RMS (106, 107), although the ability of RMS to activate HR-mediated DDR is not excluded (108, 109). On the benchside, RMS biopsies overexpressing PARP1, PARP2, and PARP3 mRNAs compared with normal skeletal muscle and PARPi have been demonstrated to affect growth, survival, and radiation susceptibility of human ARMS and ERMS cell lines (110, 111). However, on the bedside, clinical trials testing PARPi on sarcomas, not including RMS, failed (112, 113), whereas a recent phase I trial (NCT02787642) combining the PARPi with RT in locally advanced/unresectable STS, including RMS, is going to give encouraging downstaging and survival rates (114). Therefore, using PARPi could radiosensitize RMS independently of HRD or HRP phenotype because conventional RT, causing thousands of SSBs, would saturate the HR mechanisms inducing, in the presence of PARPi, RMS death, as already shown for other cancer types (115). Another potential target to affect ERMS radiosensitivity is c-Myc, whose downregulation through the inhibition of the MEK/ERK pathway has been demonstrated to *in vitro* and *in vivo* cause cell death by promoting the radiation-induced DNA DSB damage and impairing the DNA DSB repair machinery (116). NHEJ is the major DDR pathway activated by RT (117). Unlike HR, NHEJ re-ligates two broken DNA strands mainly through DNA-dependent Protein Kinase catalitic subunit (DNA-PKcs) that, complexing with Ku70/Ku80 heterodimer and DNA polymerase μ (Pol μ) and Pol λ, and in collaboration with XRCC4, XLF, LIG4, and PAXX, orchestrates this prone-error repair process. Moreover, DNA-PKcs has been shown to interplay with HR pathway, suggesting its pleiotropic role in regulating DDR (118). Targeting DNA-PKcs has been supposed to be a critical radiosensitizing strategy (89, 119), and, nowadays, several inhibitors, with a high selectivity and a valid pharmacokinetics, are available (118), including peposertib that is investigated by several clinical trials, in combination with RT or CHT plus RT, across a variety of cancer types (NCT02516813, NCT02316197, NCT03770689, NCT04555577, NCT04533750, and NCT03907969). Preclinical evidence shows that inhibiting DNA-PKcs sensitizes sarcoma to RT (120, 121), although no RMS cells have been investigated. However, several studies suggest a role for DNA-PKcs in RMS radioresistance. Specifically, DNA-PKcs has been shown to promote sarcomagenesis (122) and to sustain the activity of c-Myc (123) and AKTs (124), which are known to foster radioresistance in ERMS (116, 125–127) and ARMS tumors (128). Thus, it seems unlikely that DNA-PKcs targeting will not lead to an RMS radiosensitization. Notably, several molecules have been identified as upstream regulators of DDR in RMS including ERKs (126, 129), DNA methyltransferases 3A (DNMT3A) and DNMT3B (130), BET proteins (131), ephrin-A2 (132), caveolin-1 (CAV-1) (128), nuclear factor erythroid 2–related factor 2 (NRF-2) (133), c-Myc (116), SNAI2 (134), FAK (135), androgen receptor (136), and HDAC (137–139). Thus, another strategy to target DDR could be inhibiting these upstream molecules.

### RT and antioxidant response

RT mainly kills cancer cells by inducing the generation of ROS, which, in turn, represents the main induction mechanism of DSBs (140). Furthermore, the production of ROS can persist for several months after RT, thus enhancing the curative effects of treatment (141). However, cancer cells can activate an antioxidant stress response able to protect cells against ROS injury during RT exposure (142, 143). Kelch-like ECH-associated protein 1 and NRF2, respectively, inhibits and promotes the antioxidant response by upregulating the expression of downstream genes, such as peroxiredoxins (PRDXs), superoxide dismutases (SODs), catalase (CAT), and glutathione peroxidases 4 (GPx4) (144). The radiosensitizing effects of targeting antioxidant response in cancer cells shown on the benchside (145) have been recently confirmed on the bedside (146–148). ROS levels are critical for RMS homeostasis (149), and their modulation results are critical for the response to therapies (150). Irradiated RMS upregulates NRF2, SODs, CAT, and GPx4 expression, whereas NRF2 silencing counteracts RMS radioresistance by increasing DSBs and impairing DDR (133). Furthermore, we have recently shown that CAV-1, a tumor promoter sustaining rhabdomyosarcomagenesis (151–153), promotes radioresistance in RMS through increased oxidative stress protection (128) and that RMS surviving to RT more efficiently detoxifies from ROS (109). Increasing oxidative stress has been shown to efficiently kill RMS (154). RMS antioxidant response is finely regulated by molecular epigenetic mechanisms (137–139), known to be critical regulator of adaptive responses to stress (155, 156), including RT (157). Interestingly, the ability of RMS to detoxify from ROS increases in parallel with the acquisition of a more radioresistant phenotype (109), suggesting that, for these cells, ROS detoxification is critical to survive to radiation. However, clinical trials working by inducing ROS levels (158–160) have not included RMS. No preclinical and/or clinical data related to the use of directly targeting redox proteins drugs have been collected on RMS. However, several pieces of evidence suggest the use of drugs able to increase ROS beyond targeting redox proteins (137–139). On this regard, ROS generation has been identified as a mediator of histone deacetylase (HDAC) inhibitor (HDACi)–induced cell death (161), and the combination of HDACi with RT brings to RMS radiosensitization through increased ROS accumulation (137–139).

### RT and cell death, autophagy, and senescence

**Figure 4:**
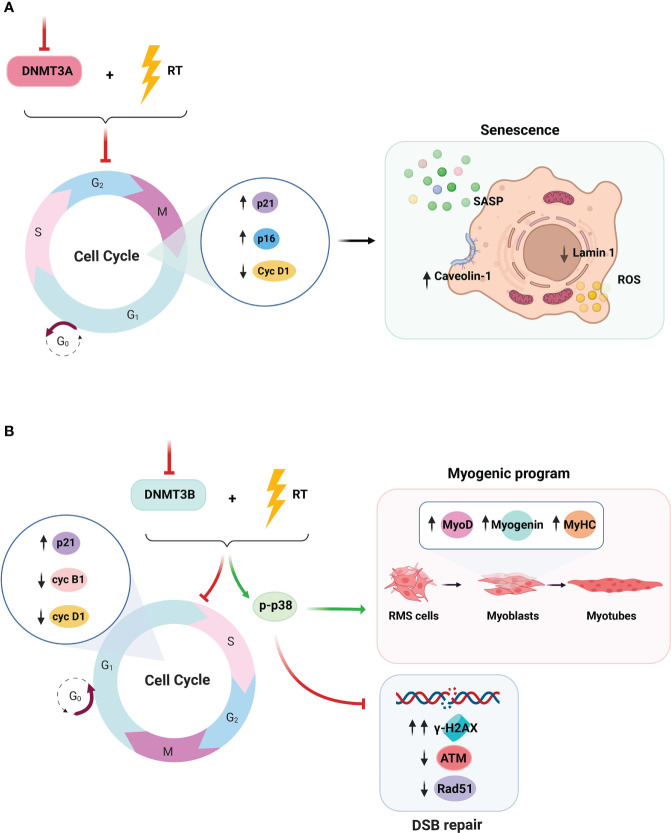
Effects of the combined treatment with DNMT3A/3B silencing and radiotherapy. Visual representation of the different radiosensitizing mechanisms observed upon **(A)** DNMT3A and **(B)** DNMT3B knocking down and RT co-treatment.

Radiobiology defines cell death as the loss of replicative capacity determined by clonogenic assays, thus including apoptosis, necrosis, mitotic catastrophe, and mitotic death, autophagy, and tumor dormancy (162, 163), although increasing evidence indicates that RT-induced tumor dormancy may not be reversible (164, 165). Apoptosis caused by RT can be mediated by the following: i) intrinsic apoptotic pathway, through the activation of the cytochrome c-caspase 9/8/3 cascade (166); ii) extrinsic apoptotic pathway, through TNF-α/TNF-R1– (167) or TRAIL/Apo2L/TRAIL–receptor–caspase 8/3 cascade (168); and iii) ceramide accumulation that, acting as second messenger, initiates a complex apoptotic program (169). Mitotic catastrophe and mitotic death, defined as the failure to undergo complete mitosis after DNA damage, coupled to defective checkpoints, are usually mediated by intrinsic apoptosis (170). In addition, cell death can be induced by inducing necroptosis, pyroptosis, and ferroptosis (162, 163). Necroptosis, mediated by TNF-α/TNF-R1/RIP1/RIP3/MLKL cascade, in the absence of caspase 8 activation (171) and pyroptosis, triggered by cytoplasmic damaged-associated molecular patterns (DAMPs) and mediated by NLRP1/NLRP3/NLRC4/caspase 1/gasdermin cascade, leads to pore formation at the cytoplasmatic membrane. Ferroptosis, induced by excessive lipid peroxidation that leads to Fe^3+^ accumulation-induced oxidative stress, is mediated by SLC11A2 and negatively regulated by GSH/GPx4 cascade (172). RMS is resistant to apoptosis (173) and necrosis (174), including from RT, as we have shown in preclinical *in vitro* and *in vivo* models (130, 137–139, 175). The tumor suppressor p53, a master promoter of apoptosis (176) and programmed necrosis (177), is frequently mutated in ERMS (178) and downregulated in ARMS (179). Recently, p53 mutations and/or pathway alterations have been associated with the increase of RMS radioresistance (180). Furthermore, RMS expresses high levels of anti-apoptotic Bcl-2 family members (181) and inactivation of caspase 8 expression by hypermethylation (182–184). On the other hand, RMS has been shown to differently modulate the expression of several factors, restraining the activation of programmed necrosis (185–188), whereas a programmed necrosis–related gene signature has been recently identified as novel prognostic biomarker for sarcoma (189). Thus, altogether, these alterations could explain the RMS resistance to RT-induced apoptosis and necrosis. Targeting cell death pathways regulating molecules has been supposed to be an opportunity for the development of innovative treatment strategies also in RMS (190). Targeting TRAIL (184, 191) and Bcl-2 (192) and reactivating caspase 8 expression (183) have been shown to promote apoptosis in RMS, alone or in combination with cytotoxic agents. The depletion of endogenous GSH by sorafenib has shown encouraging result *in vitro* and *in vivo* (193) but failed on the bedside (37), whereas others GSH inhibitors have shown anti-RMS therapeutic potential (194, 195). No data have been collected on combining pro-apoptotic or pro-necrotic agents with RT in treating RMS; however, our group has recently showed that pre-treatment with the BET inhibitor (BETi) OTX015 radiosensitizes RMS cells by inhibiting DDR and concomitantly inducing cell death as demonstrated by the strong activation of the apoptotic marker cleaved PARP (131). Death is not the only response from irradiated cells. Autophagy is a catabolic pathway for lysosomal-mediated cellular components degradation, basally inhibited by the mammalian target of rapamycin (TOR) complex 1 (mTORC1) pathway and tightly regulated by autophagy-related proteins (196). Physiologically considered as a cell survival mechanism that can also promote cell death (197), autophagy plays dual roles in cancer (198), including in RMS (199–204). Similarly, RT-induced autophagy has been shown to be cytoprotective or not (205, 206). However, increasing evidence suggests that autophagy cannot be restricted to a single cytoprotective or cytotoxic function, although it is more correct to speak about “autophagic switch”. Thus, autophagy can switch its function even within the context of a specific cancer type and/or with the respect to external stress type (207). Notably, RMS aberrantly expresses guanine nucleotide exchange factor T (208) recently shown to protects cells by inhibiting autophagy and apoptosis (204). Thus, the reduction in autophagy, which we recently shown on irradiated RMS (175), could be a mechanism of radioresistance. There is a lack of evidence on the effects of combining autophagy and RT promoters or inhibitors in the treatment of RMS. Senescence is classically defined as an irreversible form of growth arrest, mainly induced by p53, p21^WAF1^, p27^KIP1^, and p16^INK4A^ and the inhibition of cyclin-dependent kinases and RB (209). The induction of senescence represents a therapeutic advantage, thus preventing further proliferation. However, senescent cells can escape from the irreversible growth arrest status and re-enter the cell cycle, boosting tumor growth (210). In particular, these “post-senescent” cells retain stem cell–related features, also known as senescence-associated stemness, suggesting a more aggressive behavior and favoring tumor relapse (210). Senescence represents the most common cellular response after RT (211–213). However, it has been recently shown that RT-induced accumulation of senescent cells can interfere with the therapy and encourage tumor regrowth (211). Thus, the use of senolytics, small molecules that can selectively induce apoptosis of senescent cells, has been supposed to be a valid radiosensitizing strategy (214). The role of RT-induced senescence in RMS is under investigation. Our group has recently shown that DNMT3A promotes radioresistance of RMS by restraining RT-induced senescence (130), probably for the ability of DNMTs to promote DNA repair activity (215), as summarized in **Figure 4**. Furthermore, we have also found that the expression of CAV-1 protects against RT-induced cell senescence (128), thus indicating the modulation of specific regulators of cellular senescence as a promising tool to set up new and effective therapeutic intervention against RMS, mainly for overcoming tumor radioresistance-related mechanisms.

### RT and immune response

Immunotherapies, largely used in several cancer types, resulting effective in a significant fraction of standard therapy refractory patients (216), have not shown objective response in patients with RMS (217, 218). Sarcoma and particularly RMS are considered “cold” to underline the immunologically inert nature of these tumors. Thus, identifying new strategies to turn “cold” in “hot” tumors could be the way to induce immunogenic cell death (ICD) and promote the use of immunotherapies for treating sarcoma (219). RT has been shown to convert malignant cells into endogenous anticancer vaccines, thus resulting in the main strategy able to trigger ICD and boost immunotherapies. RT promotes the release of DAMPs, triggering the chemotaxis of antigen-presenting cells (APCs), dendritic cells, macrophages, and B cells, finally determining cross priming of CD8+ effector T cells. Parallelly, RT-induced cell death modifies the tumor microenvironment through the cytokine release and the expression on endothelium of cell adhesion molecules. In response, cancer cells can rapidly trigger anti-immune response by different signals including PD1/PD-L1 and CTLA4/B7-1 or B7-2 (220, 221), resulting in an opportunity for combining immunotherapies (222). However, cancer cells surviving to RT express mutated genes, increasing the presentation to APCs of neoantigens potentially able to refresh the immune response (223). This process could lead to an “antigen recycling” potentially able to re-boost the ICD-induced anticancer immune response. In this context, repeated exposure to tumor antigens released by “pulsed-RT” has been recently shown to amplify the adaptive immune response by expanding the tumor-specific T-cell receptor repertoire, the production of high-affinity tumor antibodies, and the generation of memory lymphocytes and thereby improve immune control of systemic disease (224). Therefore, the dose and fractionation seem to be the variables mainly affecting pro-immunogenic effects of RT (225, 226). Actually, HFRT and SBRT seem to improve the ability of RT to promote immune responses to tumors (225, 226). A very small percentage of RMS or RMS-infiltrating immune cells express PD-L1, more frequently in low-stage RMS and related to an increased 5-year overall survival rate (227–229). The use of immunotherapies for the treatment of pediatric advanced solid tumors has been limited (227–229), and no patients with RMS have been included. We have recently shown that Transforming growth factor beta (TGF-β), Macrophage migration inhibitory factor (MIF), C-C Motif Chemokine Ligand 2 (CCL2), C-X-C motif chemokine 5 (CXCL5), CXCL8, and CXCL12, are key players of both intrinsic and acquired radioresistance in RMS (109). Thus, targeting these cytokines, known to be mediators of radioresistance (230), could be another strategy to radiosensitize RMS tumors.

### RT and cancer stem cells

An important evidence of cancer management is the impact of RT-mediated strategies on CSCs, which are characterized by a slowly dividing subpopulation of tumoral cells capable of self-renewal features that have a critical role in tumor maintenance and metastasis as well as in resistance phenomena to conventional treatments in many cancer types (231). Recent evidence suggests that CSCs of several malignancies, also comprehending RMS, can resist ionizing radiation because of their peculiar metabolic status, associated with high expression of genes and pathways related to stem-like features, activated DNA repair mechanisms (232), and altered levels of free radical scavenger levels (233). Specifically, several studies have demonstrated the specific molecular pathways contributing to the CSC intrinsic radioresistance, such as PI3K/Akt/mTOR and NOTCH ones (234–237), which upregulates ROS scavenging enzymes (238). Thus, inhibiting NOTCH could be the efficient strategy to radiosensitize CSCs, bypassing their ability to detoxify from ROS. To date, clinical trials testing NOTCH inhibitors have not included RMS tumors as well as the combination with RT, but their use as radiosensitizers has been recently encouraged (239). More recently, boron neutron capture therapy and carbon-ion particle therapy have been proposed in combination with PARPi as effective strategies for the treatment of radioresistant clear cell sarcoma and osteosarcoma, opening up the possibility of successfully treating patients with RMS by combined treatment with RT and PARPi.

### RT and epigenetic remodeling.

Epigenetic alterations, mainly DNA methylation and histone modifications, characterize various cancers (240), including RMS (8, 241, 242). This evidence raises the question about whether the regulation of DNA methylation activity might represent a useful target for radiation sensitization. Targeting epigenetic molecules may therefore be significant in development of novel therapies, including the development of radiosensitizers (243, 244). Notably, deregulated epigenetic mechanisms have been shown to sustain different mechanisms of radioresistance including DNA repair (245, 246), antioxidant response (247, 248), cancer cell life and death decisions (249), as well as anti-cancer immune response (250). Therefore, identifying the molecules and epigenetic reprogramming pathways used by cancer cells could lead to the development of promising targeted therapies able of weakening the different mechanisms of radioresistance, also in the context of RMS. Indeed, targeting specific DNMTs or HDACs has been demonstrated to reverse RMS phenotype, counteracting stemness and inducing radiosensitization (137–139). Specifically, our group has recently demonstrated the overexpression of DNMT3A and DNMT3B in ERMS primary tumor biopsies (251) and highlighted the synergic impact of DNMT3A or DNMT3B silencing and irradiation on viability and aggressiveness of RMS cells, suggesting that DNMT inhibitors could have a clinical application in combination with standard RT in the clinical management of patients with RMS. Other strategies to reduce radioresistance-mediated mechanisms have been recently shown in ARMS tumor, the most aggressive type of RMS (137, 252). The BETi OTX015, an orally drug able to bind and block histones’ acetylated lysines, can downregulate GNL3 gene, encoding for the G nucleolar 3 protein, which is overexpressed in different malignancies, and it has been associated with uncontrolled proliferation, inhibition of programmed cell death, and resistance to therapies. Interestingly, our preclinical data also indicate that OTX015 exposure can enhance the radiosensitivity of ARMS cells by inducing a drastic G2 cell cycle arrest, which was correlated to a permanent DNA damage (upregulation of γ-H2AX) and to the inability of tumoral cells to repair it (alteration of RAD51, ATM, and DNA-PK protein expression). Moreover, OTX015 and irradiation (IR) synergistically downregulated the expression of GNL3 gene, thus suggesting a potential role of BETi in reducing cell cycle progression and maintenance of cell stemness with the potential to counteract the radioresistance phenomena. Similar remarkable radiosensitizing effects were exerted on FP-RMS cells by targeting class I and IV HDACs through MS-275 *in vitro* and *in vivo* treatment (137), confirming the crucial role of epigenetic deregulation in RMS onset and progression. Interestingly, the immunological effects of epigenetic modifiers could be used for stimulating therapeutically relevant anticancer immunity when used as stand-alone treatments or in combination with established immunotherapies, favoring the RT-induced presentation of new antigens. Thus, it is possible to assume that the antigenic recycling induced by pulsed radiotherapy, for example, could be further enhanced in presence of an epigenetic remodulation and that, therefore, the tumor can be, in this way, more easily “heated”. To this date, growing evidence suggests that radiation exposure is also related to substantial epigenetics changes of cancer cells (253). Different studies demonstrated that RT could affect DNA methylation patterns and promote a decrease in the expression level of DNMTs (254), with that genomic hypomethylation resulting in enhanced radiation sensitivity in colon carcinoma (255).

## Discussion

Years of oncology research have demonstrated the great ability of cancer cells to adapt to various therapies, including RT. Technological advances in delivering radiation have improved tumor targeting, limiting radiation exposure of healthy tissues. Thus, nowadays, the largest part of cancer patients receives RT, which results to be curative in the most cases. RT is integrated into the primary treatment of most patients with RMS. However, despite more than 90% of children with non-metastatic RMS achieves complete remission, up to one-third of them experiences a recurrence, whereas the outcome of adult patients treated with RT has not been improved. Thus, RMS remains a very deadly cancer. The use of HFRT, based on single larger dose fractions, has not led to the desired results, suggesting that the improvement of the therapeutic potential of RT goes far beyond the question of the dose, requiring knowledge and counteracting of the molecular mechanisms responsible for radioresistance. Thus, radiosensitizers remain a viable option for improving the outcome of therapy in RMS. More research is necessary to fully understand the mechanisms of RMS radioresistance and improve the outcomes of patients with this deadly disease.

## Author contributions

SCa, MC, and SP: study design, data analysis, data collection, and writing; LM, FV, AP, GB, CM, SCo, and MT: data analysis and data collection; RR and AF: review and editing; FMa and FMe: review and editing, and supervision. All authors contributed to the article and approved the submitted version.

## Funding

The research leading to these results has received funding from AIRC under IG 2020 - ID. 24696 project – P.I. FMa.

## Acknowledgments

The authors are grateful to Dr. Giulia Vitali for supporting with the images’ creation performed by using BioRender.com (2020). Retrieved from https://app.biorender.com/biorender-templates.

## Conflict of interest

The authors declare that the research was conducted in the absence of any commercial or financial relationships that could be construed as a potential conflict of interest.

## Publisher’s note

All claims expressed in this article are solely those of the authors and do not necessarily represent those of their affiliated organizations, or those of the publisher, the editors and the reviewers. Any product that may be evaluated in this article, or claim that may be made by its manufacturer, is not guaranteed or endorsed by the publisher.
